# Sequence of same‐day upper and lower gastrointestinal endoscopy does not affect total procedure' time or medication use: A randomized trial

**DOI:** 10.1002/jgh3.12203

**Published:** 2019-08-06

**Authors:** Muhammad B Hammami, Kavya M Reddy, Pratik Pandit, Elie J Chahla, Nabeel Koro, Matthew J Schuelke, Christine Hachem

**Affiliations:** ^1^ Division of Gastroenterology and Hepatology, Department of Internal Medicine Saint Louis University School of Medicine St. Louis Missouri USA; ^2^ Department of Internal Medicine Saint Louis University, School of Medicine St. Louis Missouri USA; ^3^ Research Strategy Group Saint Louis University, Office of the Vice President for Research St. Louis Missouri USA

**Keywords:** colonoscopy, digestive system endoscopy, endoscopy

## Abstract

**Background and Aim:**

Same‐day double upper and lower gastrointestinal endoscopy is frequently performed due to overlapping indications. However, it is unclear whether an upper–lower (U‐L) or lower–upper (L‐U) sequence is optimal. We analyzed the effect of sequence on total procedure time and sedation use.

**Methods:**

A total of 100 patients scheduled for same‐day double endoscopy were randomized to the U‐L or L‐U sequence arm. Primary outcomes, mean total procedure time, and sedative dosages were compared using a *t*‐test. We also explored associations of the primary outcomes with patient‐related and procedure‐related factors.

**Results:**

Comparing U‐L and L‐U sequences, mean total procedure time was 41.9 (16.2) *versus* 43.0 (14.5) min (*P* = 0.73), diphenhydramine dose 5.5 (15.4) *versus* 4.5 (14.0) mg (*P* = 0.74), fentanyl dose 71.5 (119.3) *versus* 77.6 (164.02) μg (*P* = 0.83), midazolam dose 1.6 (2.5) *versus* 1.4 (2.7) mg (*P* = 0.69), and propofol dose 437.4 (351.4) *versus* 444.5 (256.0) mg (*P* = 0.91), respectively. Total procedure and upper endoscopy times were significantly longer with trainee presence (*P* = 0.0002) and shorter with conscious sedation (*P* = 0.003). Upper endoscopy time was longer with higher body mass index (*P* = 0.001), and lower endoscopy time was longer in patients with cirrhosis or chronic kidney disease (*P* = 0.002 and 0.009, respectively). Time between procedures was significantly longer in the L‐U sequence (7.4 [2.9] *vs* 5.3 [1.1] min, [*P* < 0.001]). The study had 80% power to detect an 8 min difference in total procedure time.

**Conclusions:**

The sequence of same‐day double gastrointestinal endoscopy does not affect total procedure time or medication use. Longer total procedure and upper endoscopy times were associated with trainee presence and use of conscious sedation.

## Graphical abstract

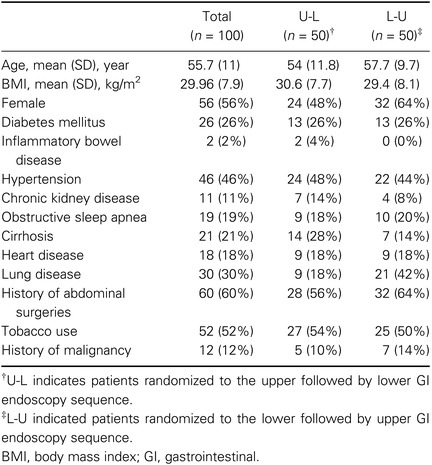

There are many benefits of same‐day bidirectional endoscopy; however, its optimal sequence (i.e. upper followed by lower or lower followed by upper) is controversial. This topic has been studied in other countries; however, this is the first study in the United States that investigated if procedure sequence affects total procedure time and sedative dose in patients undergoing same‐day upper and lower gastrointestinal (GI) endoscopy. We found that same‐day double GI endoscopy does not affect total procedure time or medication use; however, the time between procedures was longer in the colonoscopy followed by upper endoscopy group. Our results suggest that it may be reasonable to consider performing upper endoscopy first in terms of procedure order.

## Introduction

Same‐day upper and lower gastrointestinal (GI) endoscopy has been increasingly used due to overlapping indications such as evaluation of anemia, gastrointestinal bleeding without a clear source, positive stool hemeoccult test, weight loss, and patients requiring upper endoscopy for upper gastrointestinal symptoms simultaneously undergoing colon cancer screening.^1^ In the United States, a national endoscopy database showed that same‐day bidirectional endoscopy (BDE) accounts for >10% of all cases referred for gastrointestinal endoscopies.^2^ Many studies have established the benefits of same‐day upper and lower endoscopy, such as shorter hospital stays, reduced medical costs due to single‐time sedation, and fewer missed work days.^3^, ^4^ Anecdotally, many gastroenterologists and patients prefer to have both procedures performed on the same day.^5^

Although there are many benefits of same‐day BDE, its optimal sequence (i.e. upper followed by lower or lower followed by upper) is controversial. Factors involved in the selection process include the endoscopist's preference, endoscopy technician's preference, perceived efficiency issues, or depending on the way the patient is brought into the room. A few studies have examined various outcomes such as total procedure time,^6^, ^7^, ^8^, ^9^ procedure quality,^5^ patient discomfort or satisfaction,^5^, ^10^, ^11^ and total amount of sedation^6^, ^7^, ^8^, ^12^ with conflicting results. A review of the literature demonstrates eight studies, all conducted outside the United States, with variable results.^5^, ^6^, ^7^, ^8^, ^9^, ^10^, ^11^, ^12^ None of the published studies involved trainees. Some studies have suggested that upper endoscopy prior to colonoscopy may lead to a more difficult colonoscopy due to the gas insufflation required during esophagogastroduodenoscopy (EGD), while others have suggested that colonoscopy performed first can cause issues with retroflexion during EGD due to the amount of insufflated gas during colonoscopy.^5^ The aims of this study were to determine if procedure sequence affects total procedure time and sedative dose in patients undergoing same‐day upper and lower GI endoscopy in a US training hospital. We also explored potential patient‐related and procedure‐related predictors of total and individual procedure times.

## Methods

The study was performed from July 2016 to November 2017 according to the Declaration of Helsinki and after approval of the institution review board of Saint Louis University. All patients gave written informed consent.

### *Design*

This was an open‐label, randomized, single‐center study.

### *Eligibility criteria*

Adult patients who had an indication for same‐day double GI endoscopy as judged by their attending gastroenterologists were recruited. Inclusion criteria were as follows: age > 18 years and less than 90 years, scheduled upper endoscopy and a colonoscopy on the same day, use of conscious or deep sedation under anesthesia care, procedures done in endoscopy suite only, and outpatients and noncritically ill inpatients (not in the intensive care unit). Exclusion criteria included procedures not carried out on the same day, no sedation method used for any or both procedures, any or both procedures carried out outside the endoscopy suite (intensive care unit or operating room), pregnant women, and those with no decision‐making capacity. Both procedures were performed by or directly under the supervision of a board‐certified gastroenterologist with or without a gastroenterology fellow. We collected data regarding demographics, comorbidities, procedure times including total and individual times, type and amount of sedation, endoscopic findings, and adverse events. All randomized patients were analyzed.

### *Procedures*

Both upper and lower endoscopy were performed after overnight fasting, and patients consumed the standard 4 L of polyethylene glycol 3350 and electrolytes orally with instructions to split the preparation by drinking 2 L at 6 pm the night before and the other 2 L to be finished 4 h prior to the procedure time. An Olympus series 190 endoscope and colonoscope were utilized. A complete upper endoscopic exam was performed and included inspection of the esophagus, stomach with retroflexion, and exam of the duodenum (to at least the second portion). Colonoscopy was also considered complete if the base of the cecum was reached with the identification of landmarks, including the appendiceal orifice and the ileocecal valve. All procedures used room air for insufflation rather than carbon dioxide. Patients were placed in the left lateral decubitus position and were continuously monitored by the anesthesiologist, nurse anesthetist, or endoscopy nurse. Conscious sedation protocol involved giving the patient 50 μg of fentanyl and 1 mg of Midazolam as a starting dose, followed by 25 μg of fentanyl and 1 mg of midazolam every 2 min until adequate sedation was achieved. Deep sedation with the presence of an anesthesiologist typically involved giving a 30–50 mg bolus of propofol followed by the start of an infusion of propofol at 50–100 μg/kg/min. The dose and infusion rate were increased based on the patient's sedation status or movement to surgical stimulus, oxygenation, and ventilation status. Standard vitals, such as mean arterial pressure, oxygen saturation, pulse rate, and blood pressure, were monitored. After completion of the procedure, patients were monitored in the recovery unit and were not discharged until they are fully conscious.

### *Randomization*

The randomization was achieved using the website http://randomization.com (http://randomization.com). A total of 100 patients were block‐randomized to either the upper–lower (U‐L) or lower–upper (L‐U) sequence. The randomization list was concealed from potential participants and from study coordinators who recruited them.

### *Outcomes*

The primary outcome was unadjusted mean difference in total procedure time between the two arms of the study. Predetermined secondary outcomes were mean differences in medication doses. Ad hoc secondary outcomes were patient‐related and procedure‐related predictors of outcomes.

### *Sample size*

The study was designed to have 80% power to detect an 8‐min difference in total procedure time, assuming a SD of 14 and type one error of 5%. The 8‐min difference in time was based on our assumption that total time would be almost 50 min with approximately a 15% difference.

### *Statistical Analysis*

The primary and predetermined secondary outcomes were analyzed using an unpaired *t*‐test. Multiple linear regression analysis was used to explore predictors of total and individual procedure time, as well as total sedative dosages. For each outcome, the model optimizing Akaike information criterion in this dataset was selected from a full model containing all potential study predictors.

## Results

A total of 100 eligible patients were enrolled in the study and were randomly assigned, 50 to EGD followed by colonoscopy (U‐L) and 50 to colonoscopy followed by EGD (L‐U). All patients underwent both procedures, and none were excluded from analysis. The demographics of study participants are summarized in Table 1. One colonoscopy in each study arm was not completed due to patient intolerance (L‐U) and tortuous colon (U‐L). No adverse events were reported. Mean (SD) age and body mass index (BMI) were 55.7 (11) years and 30.0 (7.9) kg/m^2^, respectively. Approximately 50% of the patients were women, 60% of the patients had a history of abdominal surgery, and 11–46% suffered from various chronic diseases. Of the procedures, 63% started in the afternoon; 86% of the procedures were performed under monitored anesthesia care, and half of the procedures had a trainee present. Of the cases, 72% involved gastric biopsies and 52% involved colonic polypectomy; 52% of upper endoscopies were performed for symptoms such as pain, heartburn, dyspepsia, dysphagia, early satiety, or diarrhea followed by 39% for gastrointestinal bleeding. The most common indication for a colonoscopy (53%) was to rule out malignancy. The adenoma detection rate was 36% for colonoscopies; however, a majority of colonoscopies were not screening colonoscopies. Of the polyps, 17% were found in the ascending colon, 23% in the transverse colon, 16% in the descending colon, 25% in the rectosigmoid, and 9% in the cecum. Cecal intubation rates were 98%. Colon withdrawal times and cecum times were not obtained as they were not consistently recorded. Patient‐related and procedure‐related characteristics per study arm are summarized in Tables 2 and 3, respectively.

**Table 1:** Demographics of study participants

	Total (*n* = 100)	U‐L (*n* = 50)†	L‐U (*n* = 50)‡
Age, mean (SD), year	55.7 (11)	54 (11.8)	57.7 (9.7)
BMI, mean (SD), kg/m^2^	29.96 (7.9)	30.6 (7.7)	29.4 (8.1)
Female	56 (56%)	24 (48%)	32 (64%)
Diabetes mellitus	26 (26%)	13 (26%)	13 (26%)
Inflammatory bowel disease	2 (2%)	2 (4%)	0 (0%)
Hypertension	46 (46%)	24 (48%)	22 (44%)
Chronic kidney disease	11 (11%)	7 (14%)	4 (8%)
Obstructive sleep apnea	19 (19%)	9 (18%)	10 (20%)
Cirrhosis	21 (21%)	14 (28%)	7 (14%)
Heart disease	18 (18%)	9 (18%)	9 (18%)
Lung disease	30 (30%)	9 (18%)	21 (42%)
History of abdominal surgeries	60 (60%)	28 (56%)	32 (64%)
Tobacco use	52 (52%)	27 (54%)	25 (50%)
History of malignancy	12 (12%)	5 (10%)	7 (14%)

† U‐L indicates patients randomized to the upper followed by lower GI endoscopy sequence.

‡ L‐U indicated patients randomized to the lower followed by upper GI endoscopy sequence.

BMI, body mass index; GI, gastrointestinal.

**Table 2:** Indications for endoscopic procedure

	Total (*n* = 100)	U‐L (*n* = 50)†	L‐U (*n* = 50)‡
Upper endoscopy indication			
GI Bleeding§	39 (39%)	20 (40%)	19 (38%)
Malignancy¶	8 (8%)	5 (10%)	3 (6%)
Other symptoms††	52 (52%)	25 (50%)	27 (54%)
Lower endoscopy indication			
GI Bleeding§	21 (21%)	9 (18%)	12 (24%)
Malignancy¶	53 (53%)	30 (60%)	23 (46%)
Other symptoms††	26 (26%)	11 (22%)	15 (30%)

† U‐L indicates patients randomized to the upper followed by lower GI endoscopy sequence.

‡ L‐U indicated patients randomized to the lower followed by upper GI endoscopy sequence.

§ Indication GI Bleeding represents endoscopy performed for gastroesophageal variceal screening/surveillance, anemia, hematochezia, melena, or hematemesis.

¶ Indication Malignancy represents endoscopy performed for weight loss or Barrett's esophagus screening/surveillance.

†† Indication GI symptoms represents endoscopy performed for evaluation of celiac disease, pain, heartburn, dyspepsia, dysphagia, early satiety, or diarrhea.

GI, gastrointestinal.

**Table 3:** Procedure‐related factors

	Total (*n* = 100)	U‐L (*n* = 50)†	L‐U (*n* = 50)‡
Time of first procedure			
After noon	63 (63%)	34 (68%)	29 (58%)
Before noon	37 (37%)	16 (32%)	21 (42%)
Type of sedation			
Conscious sedation	14 (14%)	9 (18%)	5 (10%)
Monitored anesthesia care	86 (86%)	41 (82%)	45 (90%)
Presence of trainee	50 (50%)	26 (52%)	24 (48%)
Performance of gastric biopsies	72 (72%)	33 (66%)	39 (78%)
Polypectomy from ascending colon	17 (17%)	9 (18%)	8 (16%)
Polypectomy from transverse colon	23 (23%)	8 (16%)	15 (30%)
Polypectomy from descending colon	16 (16%)	6 (12%)	10 (20%)
Polypectomy from recto‐sigmoid colon	25 (25%)	11 (22%)	14 (28%)
Polypectomy from cecum	9 (9%)	6 (12%)	3 (6%)
Presence of adenomas on pathology	36 (36%)	15 (30%)	21 (42%)

† U‐L indicates patients randomized to the upper followed by lower GI endoscopy sequence.

‡ L‐U indicated patients randomized to the lower followed by upper GI endoscopy sequence.

GI, gastrointestinal.

### *Procedure time*

Time between procedure was significantly longer in the L‐U sequence, 7.4 (2.9) *versus* 5.3 (1.1) min (*P* < 0.001). Mean total procedure time was 41.9 (16.2) min in the U‐L arm and 43.0 (14.5) min in the L‐U arm (*P* = 0.73). Similarly, there were no significant differences in mean individual procedure time between the U‐L and L‐U arms, 9.8 (6.0) *versus*10 (4.4) min for EGD, (*P* = 0.82) and 26.8 (13.4) *versus* 25.6 (12.9) min for colonoscopy, (*P* = 0.64), respectively. The results are summarized in Table 4.

**Table 4:** Mean procedure times in upper–lower *versus* lower–upper groups

Mean endoscopic procedure times	Upper–lower (*n* = 50)†	Lower–upper (*n* = 50)‡	*P* (two tail)*
EGD time (SD), min	9.8 (6.0)	10 (4.4)	0.82
Colonoscopy time (SD), min	26.8 (13.4)	25.6 (12.9)	0.64
Time between (SD), min	5.3 (1.1)	7.4 (2.9)	<0.001
Total time (SD), min	41.9 (16.2)	43.0 (14.5)	0.73

* *P* value is two‐sided.

† U‐L indicates patients randomized to the upper followed by lower GI endoscopy sequence.

‡ L‐U indicated patients randomized to the lower followed by upper GI endoscopy sequence.

EGD, esophagogastroduodenoscopy; GI, gastrointestinal.

### *Medication usage*

There was no significant difference between the two study arms in the amount of any of the medications used. Mean (SD) propofol, midazolam, fentanyl, and diphenhydramine doses in the U‐L and L‐U arms were 437.4 (351.4) *versus* 444.5 (256.0) μg (*P* = 0.91), 1.6 (2.5) *versus* 1.4 (2.7) mg (*P* = 0.69), 71.5 (119.3) *versus* 77.6 (164.2) μg (*P* = 0.83), and 5.5 (15.4) *versus* 4.5 (14.0) mg (*P* = 0.74), respectively. The results are summarized in Table 5.

**Table 5:** Mean sedative doses in upper–lower *versus* lower–upper groups

Mean endoscopic sedative doses	Upper–lower (*n* = 50)†	Lower–upper (*n* = 50)‡	*P* (two tail)*
Total diphenhydramine (SD), mg	5.5 (15.4)	4.5 (14.0)	0.74
Time fentanyl (SD), μg	71.5 (119.3)	77.6 (164.02)	0.83
Time midazolam (SD), mg	1.6 (2.5)	1.4 (2.7)	0.69
Total propofol (SD), mg	437.4 (351.4)	444.5 (256.0)	0.91

* *P* value is two‐sided.

† U‐L indicates patients randomized to the upper followed by lower GI endoscopy sequence.

‡ L‐U indicated patients randomized to the lower followed by upper GI endoscopy sequence.

GI, gastrointestinal.

### *Predictors of procedure time*

Total procedure time was 13.6 min longer when a trainee was present (*P* = 0.0002), 18.5 min shorter with conscious sedation (*P* = 0.003), and 11.5 min shorter when the indication for colonoscopy was GI bleeding (*P* = 0.002). Procedure sequence continued to be insignificantly associated with total procedure time even after controlling for its other potential predictors (*P* = 0.95). EGD time was 0.21 min longer for every additional BMI unit (*P* = 0.001), 3.44 min longer for those with a history of gastric bypass surgery (*P* = 0.002), 3.48 min longer when a trainee was present (*P* = 0.007), 7.69 min shorter with conscious sedation (*P* = 0.0005), and 3.87 min shorter in patients with obstructive sleep apnea (*P* = 0.004). Procedure sequence did not significantly contribute to upper procedure time (*P* = 0.46).

Colonoscopy time was 10.36 min longer in patients with chronic kidney disease (*P* = 0.002), 8.49 min longer in patients with cirrhosis (*P* = 0.009), 1.67 min longer for every additional polyp identified on colonoscopy (*P* < 0.001), and 7.1 min longer in patients with adenomatous polyps (*P* = 0.004). Procedure sequence, trainee presence, and sedation type did not significantly contribute to longer procedure time (*P* = 0.11–0.73).

### *Predictors of medication use*

Total propofol use was 210.61 mg more in patients with chronic kidney disease (*P* = 0.008), 187.15 mg for patients with a history of gastric bypass surgery (*P* = 0.001), and 53.51 mg for every additional polyp Identified on colonoscopy (*P* < 0.001). It was less than 172.72 mg for patients with a history of abdominal surgery (*P* = 0.001), less than 265.64 mg when indication for upper endoscopy was malignancy (*P* = 0.007), and less than 285.6 mg for patients with at least one polyp in the ascending colon (*P* = 0.0002). Total midazolam use was 2.87 mg more when at least one polyp was removed from the ascending colon (*P* = 0.0001). It was less than 1.65 mg for patients with a history of gastric bypass surgery (*P* = 0.004). Total fentanyl use was more than 74.24 μg for patients with history of abdominal surgery (*P* = 0.007) and 199.49 μg for patients with at least one polyp in the descending colon (0.0001). It was less than 20.12 μg for every additional polyp identified on colonoscopy (*P* = 0.001). Total diphenhydramine use was less than 0.42 mg for every additional unit of age (*P* = 0.005) and less than 10.56 mg for patients with a history of gastric bypass surgery (*P* = 0.001).

### *Subgroup analysis of monitored anesthesia care patients*

In comparing the two study arms among the 86 patients who received deep sedation, there was no significant difference in individual EGD or colonoscopy procedure times, as well as total procedure time. However, there was a difference in time between procedures, with the U‐L group having significantly less times in between procedures (*P* < 0.001). Furthermore, there was no significant difference in total medication or doses used in this group of patients between the two study arms. Results are summarized in Table 6.

**Table 6:** Subgroup analysis of monitored anesthesia care patients: (*n* = 86)

	Upper–lower (*n* = 41)†	Lower–upper (*n* = 45)‡	*P* (two tail)*
Mean endoscopic procedure times			
Total EGD time (SD), min	8.9 (4.3)	9.6 (4.0)	0.47
Total colonoscopy time (SD), min	26.3 (12.2)	25.8 (13.0)	0.84
Time between procedures(SD), min	5.0 (1.8)	7.2 (2.5)	<0.001
Total procedure time (SD), min	40.1 (14.1)	42.5 (14.6)	0.46
Mean endoscopic sedative doses			
Total diphenhydramine (SD), mg	0.0 (0)	1.1 (7.5)	0.32
Time fentanyl (SD), μg	533.4 (314.3)	493.9 (219.2)	0.50
Time midazolam (SD), mg	0.6 (0.9)	0.7 (1.5)	0.73
Total propofol (SD), mg	533.4 (314.3)	493.9 (219.2)	0.50

* *P* value is two‐sided.

† U‐L indicates patients randomized to the upper followed by lower GI endoscopy sequence.

‡ L‐U indicated patients randomized to the lower followed by upper GI endoscopy sequence.

EGD, esophagogastroduodenoscopy; GI, gastrointestinal.

## Discussion

The aims of the study were to determine if procedure sequence affects total procedure time and medication use in patients undergoing same‐day upper and lower GI endoscopy at a tertiary care university hospital in the United States. We also explored potential patient‐related and procedure‐related predictors of total and individual procedure times. We found that: (i) procedure sequence has no significant effect on total or individual procedure times, despite significantly longer time between procedures in the L‐U sequence; (ii) procedure sequence has no significant effect on total medication used for sedation; (iii) trainee presence predicted longer total procedure time and upper endoscopy but not lower endoscopy time; and (iv) conscious sedation predicted shorter total procedure time for upper endoscopy but not lower endoscopy time.

Our results confirm and complement the results of a randomized Israeli study on 163 patients that found no significant effect of procedure sequence on patient discomfort, satisfaction, or total amount of sedation used.^1^ In addition, a Korean study also found no effect on the quality and performance of procedures in 1103 patients who had dual procedures on the same day.^10^ Furthermore, a Taiwanese study found no effect on procedure time in 176 patients who underwent dual same‐day procedures.^7^ The Israeli and Korean studies, however, did not examine total procedure time.^9^, ^10^ On the other hand, two randomized studies in Taiwan found that the U‐L sequence under moderate sedation and carbon dioxide insufflation was associated with lower sedation doses (fentanyl and midazolam) and shorter recovery time.^6^, ^8^ The Korean study suggested less subjective discomfort in the U‐L arm.^10^ In addition, a Chinese study suggested less cardiovascular stress, decreased need for sedation with propofol, and faster recovery in the U‐L sequence.^12^ A different Korean study on 80 patients found a significantly better quality of EGD performance (retroflexion‐related steps, visualization of the angular fold, and general assessment of the stomach and upper GI tract) in the U‐L arm,^5^ thought to be due to gastric distention and altered bowel motility caused by insufflated gas during colonoscopy.

As expected, we found longer total procedure time (13.6 min) when a trainee was present. Interestingly, this was almost completely due to a longer upper endoscopy time. It is not clear why trainee presence did not affect lower endoscopy time. One possible explanation is that the attending gastroenterologist may have a lower threshold to take over a colonoscopy due to procedure difficulty compared to upper endoscopy due to time constraints. We found that the total procedure time was shorter by 18.51 min with conscious sedation compared to monitored deep anesthesia (*P* = 0.003). This is likely due to improved overall health status of patients undergoing conscious sedation and likely undergoing less complicated procedures. Other explanations could include that the nurse anesthetist took more time for deep sedation cases or the endoscopy faculty may be more likely to take over the procedure from the fellow during deep sedation cases. However, it is also unclear why the effect of the type of sedation was restricted to the duration of upper endoscopy. The significant increase of in‐between time in the L‐U arm is likely related to longer times required to clean the endoscopy station after colonoscopy compared to upper endoscopy. In our endoscopy unit, if a lower endoscopy is performed first, the endoscopy station must be wiped down completely before the upper endoscopy is performed on the same patient. Per our endoscopy unit protocol, no wipe down of the endoscopy unit is required when the upper procedure is carried out first, followed by lower endoscopy in the same patient. However, interestingly, this did not translate into significantly longer total procedure time in the L‐U arm.

### *Study limitations*

The study was open label as patients and endoscopists could not be blinded. Despite the end‐points being objective, this may have affected endoscopist behavior. This was a single‐center study and due to variations in facility‐specific protocols, the results may not be generalizable to other settings. Finally, our study outcomes were restricted to procedure time and medication use. Issues such as patient satisfaction and procedure quality were not addressed. The procedure time was affected by fellows being present as this added more time. Furthermore, we did not use carbon dioxide insufflation, and this may have also affected our outcomes.

### *Conclusions*

We conclude that the sequence in same‐day double GI endoscopy does not affect total procedure time or medication use. Longer total procedure and upper endoscopy, but not lower endoscopy, times were associated with trainee presence and conscious sedation. Our study demonstrated shorter in‐between times, and other studies have found better patient satisfaction with the U‐L sequence. Thus, it may be reasonable to consider performing upper endoscopy first as the optimal sequence. Nevertheless, patient and endoscopist preferences should continue to dictate the sequence of procedures in double same‐day procedures.
